# The Impact of *PNPLA3* rs738409 Genetic Polymorphism and Weight Gain ≥10 kg after Age 20 on Non-Alcoholic Fatty Liver Disease in Non-Obese Japanese Individuals

**DOI:** 10.1371/journal.pone.0140427

**Published:** 2015-10-20

**Authors:** Kenichi Nishioji, Naomi Mochizuki, Masao Kobayashi, Mai Kamaguchi, Yoshio Sumida, Takeshi Nishimura, Kanji Yamaguchi, Hiroshi Kadotani, Yoshito Itoh

**Affiliations:** 1 Health Care Division, Kyoto Second Red Cross Hospital, Kyoto, Kyoto, Japan; 2 Molecular Gastroenterology and Hepatology, Graduate School of Medical Science, Kyoto Prefectural University of Medicine, Kyoto, Kyoto, Japan; 3 Department of Psychiatry, Shiga University of Medical Science, Otsu, Shiga, Japan; Chiba University, Graduate School of Medicine, JAPAN

## Abstract

Non-alcoholic fatty liver disease (NAFLD) in non-obese individuals is inadequately elucidated. We aim to investigate the impact of known genetic polymorphisms on NAFLD and the interaction between genetic risks and weight gain on NAFLD in obese and non-obese Japanese individuals. A total of 1164 participants who received health checkups were included. Participants with excessive alcohol consumption, with viral hepatitis or other inappropriate cases were excluded. Fatty liver was diagnosed by ultrasonography. Participants with a body mass index (BMI) of <18.5 kg/m^2^, 18.5–22.9 kg/m^2^, 23.0–24.9 kg/m^2^ and ≥25 kg/m^2^ were classified underweight, normal weight, overweight and obese, respectively. Self-administered questionnaire for lifestyle was assessed and a total of 8 previously reported genetic polymorphisms were chosen and examined. In all, 824 subjects were enrolled. The overall prevalence of NAFLD was 33.0%: 0% in underweight, 15.3% in normal weight, 41.1% in overweight and 71.7% in obese individuals. The prevalence of NAFLD is more affected by the G allele of patatin-like phospholipase domain-containing protein 3 (*PNPLA3*) rs738409 in normal weight (odds ratio (OR) 3.52; 95%-CI: 1.42–8.71; *P* = 0.0063) and in overweight individuals (OR 2.60; 95%-CI: 1.14–5.91; *P* = 0.0225) than in obese individuals (not significant). Moreover, the G allele of *PNPLA3* rs738409 and weight gain ≥10 kg after age 20 had a joint effect on the risk of NAFLD in the normal weight (OR 12.00; 95% CI: 3.71–38.79; *P* = 3.3×10^−5^) and the overweight individuals (OR 13.40; 95% CI: 2.92–61.36; *P* = 0.0008). The G allele of *PNPLA3* rs738409 is a prominent risk factor for NAFLD and the interaction between the *PNPLA3* rs738409 and weight gain ≥10 kg after age 20 plays a crucial role in the pathogenesis of NAFLD, especially in non-obese Japanese individuals.

## Introduction

Non-alcoholic fatty liver disease (NAFLD) is associated with the pathogenesis of nonalcoholic steatohepatitis (NASH), liver cirrhosis and hepatocellular carcinoma and accompanies metabolic syndromes such as type 2 diabetes (T2D), hypertension and dyslipidemia[1]. The prevalence of NAFLD increases in proportion to obesity, but in reality, this disease is not uncommon in non-obese patients in East Asia [2, 3].

Recently, several reports have shown that the prevalence of NAFLD in a non-obese (BMI< 25.0) population was 18.4% in Japan [4], 12.6% in South Korea [5], and 7.2% in China [6]. Most recently, we also reported that the prevalence of NAFLD in non-obese participants at health checkups was 15.2% [7]. Surprisingly, De la Cruz et al. reported that lean patients with NAFLD have a higher overall mortality rate than overweight or obese patients with NAFLD [8]. On the other hand, a previous review has demonstrated that ethnic differences or genetic traits affect susceptibility to NAFLD [1]. By genome-wide association studies (GWAS), genes associated with NAFLD have been determined [9, 10]. Of these, genetic polymorphisms in patatin-like phospholipase domain-containing protein 3 (*PNPLA3*) rs738409 were shown to be specifically associated with NAFLD [9, 10]. In East Asia, these polymorphisms have also been demonstrated to be associated with NAFLD [11, 12].

Additionally, several gene variants other than those in *PNPLA3* rs738409 have been regarded as contributors to NAFLD [10]. While NAFLD in non-obese East Asians, including Japanese, is striking compared with other ethnic groups, the impact of genetic factors on non-obese NAFLD has not been clearly investigated. Moreover, a routine health checkup in Japan typically includes a self-administered questionnaire provided by the Ministry of Health, Labor and Welfare of Japan [13]. Our previous report [7] and a recent large-scale Japanese study [14] revealed that the presence of weight gain ≥10 kg after age 20 among the lifestyle information in this questionnaire was a prominent risk factor for NAFLD even in non-obese Japanese individuals.

Therefore, an evaluation of the genetic traits and the interaction between significant genetic risk factors and weight gain ≥10 kg after age 20 with respect to the pathogenesis of NAFLD in non-obese Japanese individuals is required. Here, we conducted a cross-sectional study that included Japanese participants who received health checkups at our hospital in Kyoto, Japan.

## Materials and Methods

### Ethics statement

Prior to sample collection, written informed consent was obtained from each participant for the anonymous use of their data for an epidemiological study. The study design was approved by the institutional review board of Kyoto Second Red Cross Hospital (No.S24-18) and Kyoto Prefectural University of Medicine Ethical Review Board (No.G-129). The study was conducted in accordance with the Declaration of Helsinki.

### Study population and design

**Fig 1:**
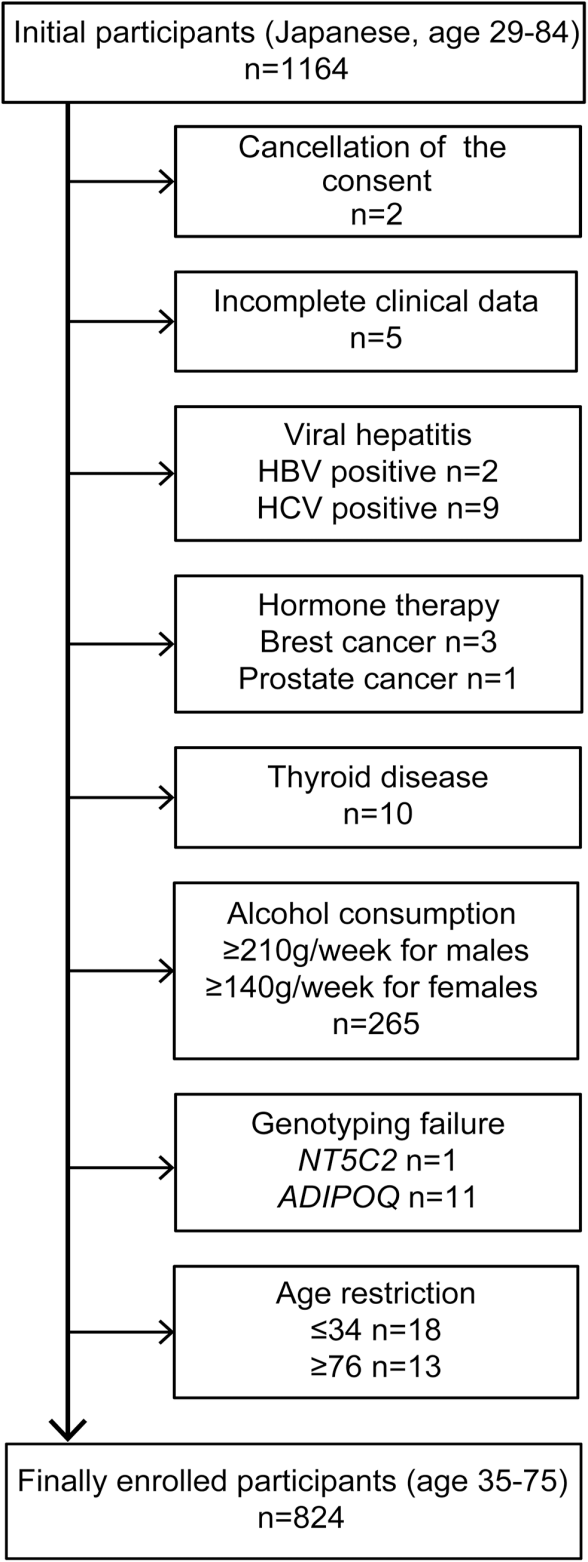
Flowchart of the inclusion and exclusion criteria. HBV, hepatitis B virus; HCV, hepatitis C virus; *NT5C2*, 5’-nucleotidase, cytosolic II; *ADIPOQ*, adiponectin.

We included 1164 Japanese individuals aged 29–84 years who participated in health checkups, including physical and physiological examinations, abdominal ultrasonography (US) and blood screening examinations, during the period of September 2012 to March 2014 at Kyoto Second Red Cross Hospital in Japan. Exclusion criteria were as follows: (1) inappropriate cases (e.g., insufficient or indeterminate data); (2) hepatitis virus infection (i.e., hepatitis B surface antigen-positive and hepatitis C virus antibody-positive); (3) the presence of autoimmune liver disease; (4) ongoing treatment for thyroid disease; (5) ongoing hormone therapy; (6) excessive use of alcohol (≥210 g/week for males and ≥140 g/week for females); (7) age ≤34 and ≥76 years. Finally, 824 participants aged 35–75 years who met the inclusion criteria were enrolled (Fig 1). According to the Western Pacific Region of the WHO criteria that pertain to obesity (WPRO criteria), the participants with a body mass index (BMI) <18.5 kg/m^2^ were classified as underweight, those with a BMI of 18.5–22.9 kg/m^2^ were classified as normal weight, those with a BMI of 23.0–24.9 kg/m^2^ were classified as overweight and those with a BMI ≥25.0 kg/m^2^ were classified as obese [15].

### Physical examination and laboratory assessments

Body weight (BW) and height (Ht) were obtained for both sets of participants, and the BMI was calculated. Waist circumference (WC) was measured by physicians at the level of the navel when the participants were in a standing position. Venous blood samples were obtained from all participants at 9AM following a 12-h overnight fast. The levels of aspartate aminotransferase (AST), alanine aminotransferase (ALT), gamma-glutamyl transpeptidase (GGT), total cholesterol (TC), high-density lipoprotein (HDL) cholesterol, low-density lipoprotein (LDL) cholesterol, triglycerides (TG), fasting plasma glucose (FPG) were measured using an automated analyzer with standard techniques. Additionally, we estimated the value of FIB4 index which is considered a useful, simple and non-invasive marker for predicting hepatic fibrosis in patients with NAFLD [16]. The value of the FIB4 index which is based on age, AST, ALT and platelet counts were calculated in accordance with the established formula. Shah et al. proposed that a low cut-off point (1.3) and a high cut-off point (2.67) of the FIB4 index are regarded as the negative predictive value and the positive predictive value for detecting stage 3–4 advanced fibrosis in patients with NAFLD, respectively [16].

Hypertension, type 2 diabetes (T2D) and dyslipidemia were diagnosed by the following criteria: hypertension was considered present in patients on medication for hypertension and/or in patients whose systolic blood pressure (SBP) was ≥140 mmHg and/or in patients whose diastolic blood pressure (DBP) was ≥90 mmHg; T2D was considered present in patients on medication for T2D and/or when the FPG was ≥126 mg/dl; dyslipidemia was considered present in patients on medication for dyslipidemia and/or when the level of LDL cholesterol was ≥140 mg/dl and/or when the level of TG was ≥150 mg/dl and/or when the level of HDL cholesterol was <40 mg/dl. According to the previous report [17], the normal range for ALT level was ≤30 U/l and ALT elevation was defined as level ≥31 U/l.

### DNA preparation and analysis

Genomic DNA was extracted from EDTA-blood from a blood sample using a DNeasy Blood & Tissue Kit (QIAGEN, Tokyo, Japan). We selected 8 single nucleotide polymorphisms (SNPs). We analyzed the genetic variations in the previously reported genes rs2228603 in neurocan (*NCAN*), rs12137855 in lysophospholipase-like 1 (*LYPLAL1*), rs780094 in glucokinase regulatory protein (*GCKR*) and rs4240624 in protein phosphatase 1, regulatory subunit 3b (*PPP1R3B*) with real-time amplification by sequence-specific primers (S1 Table) and melting curve analysis using a Light Cycler Instrument [10].

The primer pairs were used for the amplification of the *NCAN*, *LYPLAL1*, *GCKR* and *PPP1R3B* genes. The sensor probes were labeled with 3´-fluorescein, and the anchor probes were labeled with 5´-LightCycler Red-640. The PCR mixture was prepared using the Light Cycler Fast Start DNA Master Hybridization Probes (Roche Applied Science, Indianapolis, IN, USA). Genotyping for rs1004467 in cytochrome P450, family 17, subfamily A, polypeptide 1 (*CYP17A1*), rs11191548 in 5’-nucleotidase, cytosolic II (*NT5C2*), rs6810075 in adiponectin (*ADIPOQ*) and rs738409 in *PNPLA3* was completed using the TaqMan SNP genotyping assay (Applied Biosystems, Foster City, CA, USA) with commercially available pre-designed SNP-specific primers for PCR amplification and extension reactions according to the manufacturer’s protocol [9,10, 18, 19].

### Abdominal US protocol and the definition of fatty liver

All subjects received an abdominal US to assess the liver. The liver parenchyma in all participants was examined using a conventional convex array transducer. The presence of fatty change was defined as an increased echogenicity of the liver parenchyma compared with the renal parenchyma (bright liver and liver-kidney contrast), deep US attenuation in the right lobe of the liver (deep attenuation) and/or poor visualization of the hepatic vein (vascular blurring) [7, 20]. The abdominal US was performed by experienced sonographers with a Xario unit with a 3.5-MHz convex array transducer (Toshiba Medical Systems, Tokyo, Japan). The technical parameters were adjusted for each participant using the standard protocol for US. Four board-certified gastroenterologists of the Japanese Society of Gastroenterology reviewed the images and made a diagnosis.

### Questionnaire

Lifestyle was assessed using a self-administered questionnaire provided by the Health Department of the Ministry of Health, Labor and Welfare in Japan [13] that properly included information about disease history and various lifestyle habits (S2 Table). The information from the questionnaire was confirmed during an interview with a physician [7].

### Statistical analysis

The enrolled subjects were categorized into 4 groups according to BMI, as mentioned above, and were assessed [15]. Descriptive statistics (means and standard deviations) were calculated for all continuous variables. Differences between 2 groups were assessed using the Mann–Whitney *U* test or the chi square test. Differences between 3 or more groups were assessed using the chi square test with Bonferroni multiple testing correction. The Mann–Whitney *U* test was used for continuous variables, and the chi square test was used for categorical variables.

We compared the risk between specific allele carriers and non-carriers. In addition, as the A allele of *CYP17A1* rs1004467 and the T allele of *NT5C2* rs11191548 are associated with a reduced visceral fat area (VFA) and a reduced subcutaneous fat area (SFA) [18], the G allele of *CYP17A1* rs1004467 and the C allele of *NT5C2* rs11191548 were considered risk factors for VFA and SFA. The risk allele frequencies were calculated, and the Hardy-Weinberg equilibrium was examined using the chi square test. Univariate and multivariate logistic regression analyses were performed to identify the variables that are risk factors for NAFLD.

Model 1 aimed to identify the significant factors that are associated with NAFLD among the genetic polymorphisms included in this study. After adjusting for age and gender, only the significant covariates among them were calculated. Model 2 aimed to identify the independent predictors of NAFLD among these polymorphisms and clinical parameters. In addition to the age and gender, known metabolic traits such as hypertension, T2D, dyslipidemia and weight gain ≥10 kg after age 20, are strong predictors of NAFLD among the self-reported lifestyle items in line with the previous reports [7, 14]; these were therefore taken into account during the analysis. Furthermore, these genetic polymorphisms, age, gender, metabolic traits and ALT elevation (≥31 U/l) were examined in Model 3 because ALT was a significant factor for NAFLD in the univariate analysis.

Parameters with effects at *P* <0.05 were entered into the stepwise multivariate logistic regression analysis with *P* <0.05 for inclusion and *P* ≥0.05 for exclusion. Values of *P* <0.05 were considered statistically significant. The statistical analysis was performed using SPSS statistical software version 17.0 (SPSS, Chicago, IL, USA).

## Results

### Clinical features of the study population

Of the 1164 participants, 340 cases were excluded, and finally, 824 cases who fulfilled the criteria were enrolled. Of these, the mean age was 54.3 years. A total of 548 cases (66.5%) were male, 276 cases (33.5%) were female, 198 cases (24.0%) were obese and 272 cases (33.0%) were diagnosed with NAFLD. The demographic, clinical, metabolic features of these cases are summarized in S3 Table and the genetic variations of these cases are summarized in Table 1. No cases of NAFLD occurred in individuals considered underweight (BMI <18.5). The prevalence of NAFLD was 15.3% in the normal weight (BMI 18.5–22.9) group, 41.1% in the overweight (BMI 23.0–24.9) group and 71.7% in the obese (BMI ≥25.0) group.

**Table 1:** The genetic polymorphisms of NAFLD and non-NAFLD.

		Underweight (BMI<18.5) (n = 65)				
		Non-NAFLD (n = 65)				
Variables	Allele	Genotype	RA + vs—(%)			
		(RAF)				
*NCAN*	C/T*	56/9/0	86.2/13.8			
		(0.0692)				
*LYPLAL1*	C*/T	54/0/1	98.2/1.8			
		(0.9076)				
*GCKR*	C/T*	12/36/17	18.5/81.5			
		(0.5384)				
*PPP1R3B*	A*/G	63/2/0	100.0/0.0			
		(0.9846)				
*CYP17A1*	A/G*	31/26/8	47.7/52.3			
		(0.3230)				
*NT5C2*	C*/T	7/20/38	41.5/58.5			
		(0.2615)				
*ADIPOQ*	C/T*	13/33/19	20.0/80.0			
		(0.5461)				
*PNPLA3*	C/G*	20/36/9	30.8/69.2			
		(0.4153)				
		Normal weight (BMI 18.5–22.9) (n = 391)				
		NAFLD (n = 60)		Non-NAFLD (n = 331)		
Variables	Allele	Genotype	RA + vs—(%)	Genotype	RA + vs—(%)	*P*-value
		(RAF)		(RAF)		
*NCAN*	C/T*	51/9/0	85.0/15.0	297/33/1	89.7/10.3	0.2682
		(0.0750)		(0.0528)		
*LYPLAL1*	C*/T	49/11/0	100.0/0.0	294/36/1	99.7/0.3	1.0000
		(0.9083)		(0.9425)		
*GCKR*	C/T*	10/28/22	16.7/83.3	59/157/115	17.8/82.2	0.8285
		(0.6000)		(0.5845)		
*PPP1R3B*	A*/G	58/2/0	100.0/0.0	324/7/0	100.0/0.0	1.0000
		(0.9833)		(0.9894)		
*CYP17A1*	A/G*	22/32/6	36.7/63.3	162/127/42	48.9/51.1	0.0796
		(0.3666)		(0.3187)		
*NT5C2*	C*/T	5/26/29	51.7/48.3	31/122/178	46.2/53.8	0.4370
		(0.3000)		(0.2779)		
*ADIPOQ*	C/T*	18/21/21	30.0/70.0	73/180/78	22.0/78.0	0.1802
		(0.5250)		(0.5075)		
*PNPLA3*	C/G*	7/33/20	11.7/88.3	95/174/62	28.7/71.3	0.0057
		(0.6083)		(0.4501)		
		Overweight (BMI 23.0–24.9) (n = 170)				
		NAFLD (n = 70)		Non-NAFLD (n = 100)		
Variables	Allele	Genotype	RA + vs—(%)	Genotype	RA + vs—(%)	*P*-value
		(RAF)		(RAF)		
*NCAN*	C/T*	64/6/0	91.4/8.6	94/6/0	94.0/6.0	0.5543
		(0.0428)		(0.0300)		
*LYPLAL1*	C*/T	60/9/1	98.6/1.4	88/12/0	100.0/0.0	0.4117
		(0.9214)		(0.9400)		
*GCKR*	C/T*	10/34/26	14.3/85.7	16/58/26	16.0/84.0	0.7598
		(0.6142)		(0.5500)		
*PPP1R3B*	A*/G	68/2/0	100.0/0.0	100/0/0	100.0/0.0	1.0000
		(0.9857)		(1.0000)		
*CYP17A1*	A/G*	34/30/6	48.6/51.4	44/46/10	44.0/56.0	0.5560
		(0.3000)		(0.3300)		
*NT5C2*	C*/T	5/25/40	42.9/57.1	7/38/55	45.0/55.0	0.7818
		(0.2500)		(0.2600)		
*ADIPOQ*	C/T*	18/26/26	25.7/74.3	22/53/25	22.0/78.0	0.5741
		(0.5571)		(0.5150)		
*PNPLA3*	C/G*	10/38/22	14.3/85.7	34/48/18	34.0/66.0	0.0038
		(0.5857)		(0.4200)		
		Obese (BMI >25.0) (n = 198)				
		NAFLD (n = 142)		Non-NAFLD (n = 56)		*P*-value
Variables	Allele	Genotype	RA + vs—(%)	Genotype	RA + vs—(%)	
		(RAF)		(RAF)		
*NCAN*	C/T*	125/17/0	88.0/12.0	52/4/0	92.9/7.1	0.4441
		(0.0598)		(0.0357)		
*LYPLAL1*	C*/T	125/16/1	99.3/0.7	46/9/1	98.2/1.8	0.4866
		(0.9366)		(0.9017)		
*GCKR*	C/T*	21/76/45	14.8/85.2	10/33/13	17.9/82.1	0.6647
		(0.5845)		(0.5267)		
*PPP1R3B*	A*/G	139/3/0	100.0/0.0	54/2/0	100.0/0.0	1.0000
		(0.9894)		(0.9821)		
*CYP17A1*	A/G*	56/66/20	39.4/60.6	17/33/6	30.4/69.6	0.2330
		(0.3732)		(0.4017)		
*NT5C2*	C*/T	13/65/64	54.9/45.1	5/23/28	50.0/50.0	0.5310
		(0.3204)		(0.2946)		
*ADIPOQ*	C/T*	40/61/41	28.2/71.8	21/23/12	37.5/62.5	0.2002
		(0.5035)		(0.4196)		
*PNPLA3*	C/G*	37/71/34	26.1/73.9	21/26/9	37.5/62.5	0.1110
		(0.4894)		(0.3928)		

RAF, risk allele frequency; BMI, body mass index; RA + vs -, carriers of the risk allele versus non-carriers; *NCAN*, neurocan; *LYPLAL1*, lysophospholipase-like 1; *GCKR*, glucokinase regulatory protein; *PPP1R3B*, protein phosphatase 1, regulatory subunit 3b; *CYP17A1*, cytochrome P450, family 17, subfamily A, polypeptide 1; *NT5C2*, 5’-nucleotidase, cytosolic II, *ADIPOQ*, adiponectin; *PNPLA3*, patatin-like phospholipase domain-containing protein 3. The risk allele of each SNP is marked by an asterisk. *P-*values were calculated with the chi square test for categorical variables. *P-*values for the genetic polymorphisms show the difference between carriers of the risk allele and non-carriers.

Taken together, the prevalence was 20.7% in the non-obese (BMI ≤24.9) population. The prevalence was higher in males than in females in all groups. A significant difference was observed in the values of WC, AST, ALT, GGT, TG, HDL and FPG and in the proportion of weight gain ≥10 kg after age 20 between individuals with NAFLD and those with non-NAFLD, regardless of the group. The distributions of each genetic polymorphism in all participants were in Hardy-Weinberg equilibrium and were statistically similar to the distribution found in a Japanese population that was included in the International HapMap Project.

### The association between genetic polymorphisms, metabolic traits and NAFLD

By univariate analysis, crude odds ratios (ORs) of these factors in the 4 groups were determined and are shown in Table 2. TT homozygotes of *LYPLAL1* rs12137855 were very rare and there was no GG homozygote of *PPP1R3B* rs4240624. Consequently, the ORs for *LYPLAL1* rs12137855 in the normal and in the overweight groups and those for *PPP1R3B* rs4240624 in all groups were unable to be calculated statistically.

**Table 2:** The association between genetic polymorphisms, metabolic traits and the risk of NAFLD.

	Normal weight(BMI 18.5–22.9)(n = 391)	
Variables	*P*-value	Crude OR (95%CI)
Age (years)	0.1123	1.02 (0.99–1.04)
Gender (male)	0.0009	3.18(1.59–6.34)
*NCAN* [T]	0.2844	1.54 (0.69–3.40)
*LYPLAL1* [C]	-	-
*GCKR* [T]	0.8286	1.08 (0.52–2.26)
*PPP1R3B* [A]	-	-
*CYP17A1* [G]	0.0816	1.65 (0.93–2.92)
*NT5C2* [C]	0.4375	1.24 (0.71–2.15)
*ADIPOQ* [T]	0.1823	0.66 (0.35–1.21)
*PNPLA3* [G]	0.0079	3.04 (1.33–6.94)
Hypertension	0.0054	2.33 (1.28–4.24)
T2D	0.0522	2.71 (0.99–7.45)
Dyslipidemia	1.1×10^−4^	3.22(1.78–5.83)
Weight gain ≥10 kg after age 20	6.9×10^−5^	3.68 (1.93–7.00)
ALT elevation	1.1×10^−6^	6.32 (3.00–13.30)
	Overweight (BMI 23.0–24.9) (n = 170)	
Variables	*P*-value	Crude OR (95%CI)
Age (years)	0.7076	0.99 (0.96–1.02)
Gender (male)	0.0010	4.79 (1.87–12.24)
*NCAN* [T]	0.5214	1.46 (0.45–4.75)
*LYPLAL1* [C]	-	-
*GCKR* [T]	0.7599	1.14 (0.48–2.69)
*PPP1R3B* [A]	-	-
*CYP17A1* [G]	0.5562	0.83 (0.45–1.53)
*NT5C2* [C]	0.7818	0.91 (0.49–1.69)
*ADIPOQ* [T]	0.5745	0.81 (0.39–1.66)
*PNPLA3* [G]	0.0049	3.09 (1.40–6.79)
Hypertension	0.6719	0.87 (0.45–1.65)
T2D	0.3740	1.84 (0.47–7.13)
Dyslipidemia	0.0760	1.78 (0.94–3.38)
Weight gain ≥10 kg after age 20	0.0032	2.60 (1.37–4.91)
ALT elevation	0.0025	3.36 (1.53–7.37)
	Obese (BMI >25.0) (n = 198)	
Variables	*P*-value	Crude OR (95%CI)
Age (years)	0.2371	1.02 (0.98–1.05)
Gender (male)	0.0506	2.04 (0.99–4.16)
*NCAN* [T]	0.3256	1.76 (0.56–5.50)
*LYPLAL1* [C]	0.5082	2.56 (0.15–41.70)
*GCKR* [T]	0.5930	1.25 (0.54–2.86)
*PPP1R3B* [A]	-	-
*CYP17A1* [G]	0.2344	0.66 (0.34–1.29)
*NT5C2* [C]	0.5313	1.21 (0.65–2.26)
*ADIPOQ* [T]	0.2017	1.53 (0.79–2.94)
*PNPLA3* [G]	0.1129	1.70 (0.88–3.28)
Hypertension	0.0115	2.53 (1.23–5.20)
T2D	0.0467	3.05 (1.01–9.16)
Dyslipidemia	0.0262	2.03 (1.08–3.82)
Weight gain ≥10 kg after age 20	0.0084	2.88 (1.31–6.33)
ALT elevation	2.3×10^−6^	10.49 (3.95–27.83)

OR: odds ratio, CI: confidence interval.

*NCAN*, neurocan; *LYPLAL1*, lysophospholipase-like 1; *GCKR*, glucokinase regulatory protein; *PPP1R3B*, protein phosphatase 1, regulatory subunit 3b; *CYP17A1*, cytochrome P450, family 17, subfamily A, polypeptide 1; *NT5C2*, 5’-nucleotidase, cytosolic II, *ADIPOQ*, adiponectin; *PNPLA3*, patatin-like phospholipase domain-containing protein 3; T2D, type 2 diabetes. The risk allele of each SNP is shown in brackets. Metabolic traits include hypertension, T2D, dyslipidemia and weight gain ≥10 kg after age 20.

The predictors of NAFLD as determined by stepwise multivariate logistic regression analysis are shown in Tables 3, 4 and 5. In Model 1, *CYP17A1* rs1004467 and *PNPLA3* rs738409 were predictors of NAFLD in the individuals of normal weight independently of age and gender. *PNPLA3* rs738409 was a predictor in overweight individuals (Table 3).

**Table 3:** The association between genetic polymorphisms and the risk of NAFLD. (Model 1).

	Normal weight (BMI 18.5–22.9) (n = 391)	
Variables	*P*-value	Adjusted OR (95%CI)
Age (years)	0.0103	1.03 (1.00–1.06)
Gender (male)	2.4×10^−4^	3.86 (1.87–7.94)
*CYP17A1* [G]	0.0462	1.82 (1.01–3.29)
*PNPLA3* [G]	0.0073	3.15 (1.36–7.31)
	Overweight (BMI 23.0–24.9) (n = 170)	
Variables	*P*-value	Adjusted OR (95%CI)
Gender (male)	0.0023	4.38 (1.69–11.36)
*PNPLA3* [G]	0.0131	2.78 (1.24–6.26)

OR: odds ratio, CI: confidence interval.

*CYP17A1*, cytochrome P450, family 17, subfamily A, polypeptide 1; *PNPLA3*, patatin-like phospholipase domain-containing protein 3. The risk allele of each SNP is shown in brackets. Age, gender and the genetic polymorphisms were examined in Model 1.

**Table 4:** The association between genetic polymorphisms, metabolic traits and the risk of NAFLD. (Model 2).

	Normal weight (BMI 18.5–22.9) (n = 391)	
Variables	*P*-value	Adjusted OR (95%CI)
Gender (male)	0.0019	3.16 (1.52–6.55)
*PNPLA3* [G]	0.0112	3.07 (1.29–7.30)
Hypertension	0.0314	2.03 (1.06–3.87)
Dyslipidemia	5.2×10^−4^	3.02 (1.61–5.64)
Weight gain ≥10 kg after age 20	0.0059	2.62 (1.32–5.23)
	Overweight (BMI 23.0–24.9) (n = 170)	
Variables	*P*-value	Adjusted OR (95%CI)
Gender (male)	0.0052	3.94 (1.50–10.32)
*PNPLA3* [G]	0.0225	2.60 (1.14–5.91)
Weight gain ≥10 kg after age 20	0.0170	2.25 (1.15–4.40)
	Obese (BMI >25.0) (n = 198)	
Variables	*P*-value	Adjusted OR (95%CI)
Gender (male)	0.0386	2.23 (1.04–4.77)
Hypertension	0.0117	2.63 (1.23–5.58)
Dyslipidemia	0.0342	2.04 (1.05–3.96)
Weight gain ≥10 kg after age 20	0.0136	2.82 (1.23–6.45)

OR: odds ratio, CI: confidence interval.

*PNPLA3*, patatin-like phospholipase domain-containing protein 3; T2D, type 2 diabetes. The risk allele of each SNP is shown in brackets. Metabolic traits include hypertension, T2D, dyslipidemia and weight gain ≥10 kg after age 20. Age, gender, the genetic polymorphisms, hypertension, T2D, dyslipidemia and weight gain ≥10 kg after age 20 were examined in Model 2.

**Table 5:** The association between genetic polymorphisms, metabolic traits, ALT elevation and the risk of NAFLD. (Model 3).

	Normal weight (BMI 18.5–22.9) (n = 391)	
Variables	*P*-value	Adjusted OR (95%CI)
Gender (male)	0.0101	2.62 (1.25–5.49)
*PNPLA3* [G]	0.0063	3.52 (1.42–8.71)
Dyslipidemia	0.0007	2.99 (1.58–5.68)
Weight gain ≥10 kg after age 20	0.0042	2.79 (1.38–5.65)
ALT elevation	5.5×10^−5^	5.29 (2.35–11.90)
	Overweight (BMI 23.0–24.9) (n = 170)	
Variables	*P*-value	Adjusted OR (95%CI)
Gender (male)	0.0052	3.94 (1.50–10.32)
*PNPLA3* [G]	0.0225	2.60 (1.14–5.91)
Weight gain ≥10 kg after age 20	0.0170	2.25 (1.15–4.40)
	Obese (BMI >25.0) (n = 198)	
Variables	*P*-value	Adjusted OR (95%CI)
Age	0.0111	1.05 (1.01–1.09)
ALT elevation	4.3×10^−7^	13.80 (4.98–38.20)

OR: odds ratio, CI: confidence interval.

*PNPLA3*, patatin-like phospholipase domain-containing protein 3; T2D, type 2 diabetes. The risk allele of each SNP is shown in brackets. Metabolic traits include hypertension, T2D, dyslipidemia and weight gain ≥10 kg after age 20. Age, gender, the genetic polymorphisms, hypertension, T2D, dyslipidemia, weight gain ≥10 kg after age 20 and ALT elevation were examined in Model 3.

In Model 2, gender, *PNPLA3* rs738409, hypertension, dyslipidemia and weight gain ≥10 kg after age 20 were the independent predictors in the patients with normal weight. Likewise, gender, *PNPLA3* rs738409 and weight gain ≥10 kg after age 20 were independent predictors in the patients who were overweight. Gender, hypertension, dyslipidemia and weight gain ≥10 kg after age 20 were also independent predictors in obese individuals (Table 4).

In Model 3, gender, *PNPLA3* rs738409, dyslipidemia, weight gain ≥10 kg after age 20 and ALT elevation were the independent predictors in patients with normal weight. Likewise, gender, *PNPLA3* rs738409 and weight gain ≥10 kg after age 20 were independent predictors in the patients who were overweight. Age and ALT elevation were also independent predictors in obese individuals (Table 5).

In total (n = 824), independently of age and gender, *PNPLA3* rs738409 was a predictor (adjusted OR = 1.73, 95%CI: 1.20–2.48, *P* = 0.0027) in Model 1. In Model 2, weight gain ≥10 kg after age 20 (adjusted OR = 6.82, 95%CI: 4.81–9.65, *P* = 2.6×10^−27^), gender (adjusted OR = 3.12, 95%CI: 2.06–4.72, *P* = 6.9×10^−8^), dyslipidemia (adjusted OR = 2.14, 95%CI: 1.50–3.05, *P* = 2.1×10^−5^), *PNPLA3* rs738409 (adjusted OR = 1.91, 95%CI: 1.26–2.88, *P* = 0.0019) and hypertension (adjusted OR = 1.71, 95%CI: 1.18–2.50, *P* = 0.0046) were independent predictors for NAFLD. In Model 3, weight gain ≥10 kg after age 20 (adjusted OR = 5.65, 95%CI: 3.93–8.13, *P* = 9.1×10^−21^), ALT elevation (adjusted OR = 5.31, 95%CI: 3.34–8.45, *P* = 1.7×10^−12^), gender (adjusted OR = 2.60, 95%CI: 1.69–3.99, *P* = 1.3×10^−5^), dyslipidemia (adjusted OR = 1.98, 95%CI: 1.37–2.87, P = 0.0002), *PNPLA3* rs738409 (adjusted OR = 1.98, 95%CI: 1.28–3.04, *P* = 0.0019) and hypertension (adjusted OR = 1.62, 95%CI: 1.09–2.40, *P* = 0.0156) were independent predictors for NAFLD.

The values of the FIB4 index and the prevalence of an FIB4 index low cut-off index (<1.3), indeterminate (1.3–2.67) and high cut-off index (>2.67) was shown in S3 Table. Furthermore, we analyzed the association between the values of the FIB4 index and the *PNPLA3* rs738409 genetic polymorphism in patients with NAFLD. From the results, the FIB4 index was not significantly associated with *PNPLA3* rs738409 genetic polymorphism in total, in normal weight, in overweight and in obese groups, respectively (data not shown).

### The joint effect of the PNPLA3 rs738409 variant and weight gain ≥10 kg after age 20 on NAFLD

**Fig 2:**
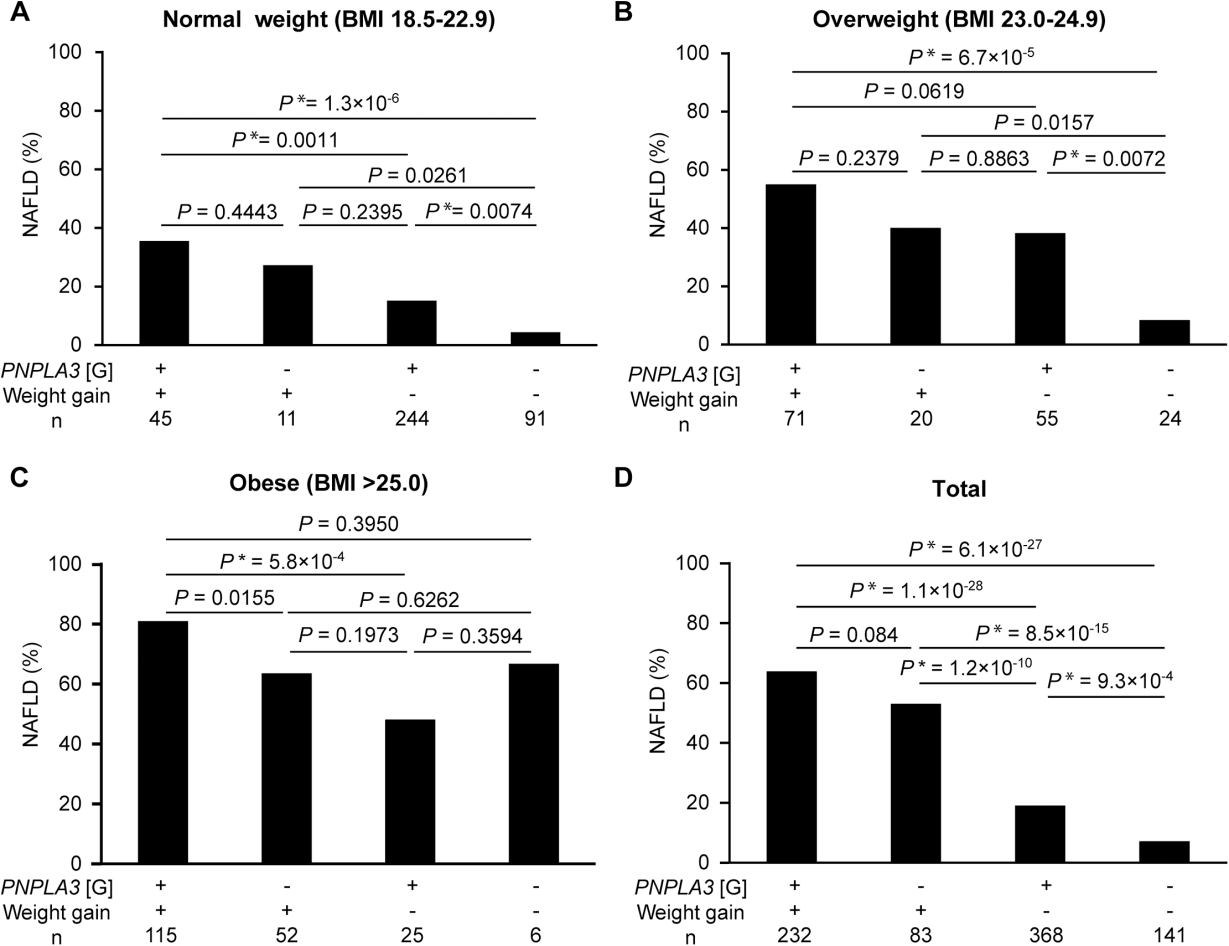
The interaction between the G risk allele in *PNPLA3* rs738409 and weight gain ≥10 kg after age 20 on the prevalence of NAFLD according to group. The normal weight group (A), the overweight group (B), and the obese group (C) are shown, respectively. The relative percentage of NAFLD is shown for each group. Differences between the groups were calculated using the chi square test. After Bonferroni multiple testing correction, values of *P* <0.0083 were considered statistically significant and were marked with an asterisk. The risk allele of *PNPLA3* rs738409 is enclosed in brackets.

The prevalence of NAFLD was significantly higher in carriers of the G risk allele in *PNPLA3* rs738409 than in non-carriers without weight gain ≥10 kg after age 20 in the normal weight and overweight groups (Fig 2A and 2B). The prevalence of NAFLD did not differ between carriers of the G risk allele and non-carriers with weight gain ≥10 kg after age 20, regardless of the group (Fig 2A–2C). The highest prevalence of NAFLD was observed in the carriers of the G risk allele with weight gain ≥10 kg after age 20 in all groups (Fig 2A–2C). The lowest prevalence was observed in non-carriers without weight gain ≥10 kg after age 20 in the normal weight and overweight groups (Fig 2A and 2B).

In addition, non-carriers without weight gain ≥10 kg after age 20 were considered the reference group, and the crude OR was assessed using logistic regression analysis. Compared with the reference group, the G risk allele carriers with weight gain ≥10 kg after age 20 had ORs of 12.00 in the normal weight group and 13.40 in the overweight group (Table 6).

**Table 6:** The association between the G risk allele in *PNPLA3* rs738409, weight gain ≥10 kg after age 20 and the risk of NAFLD.

	Normal weight (BMI 18.5–22.9) (n = 391)	
Category	*P*-value	Crude OR (95%CI)
*PNPLA3* [G]- Weight gain-	-	1.00 (Reference)
*PNPLA3* [G]+ Weight gain-	0.0121	3.88 (1.34–11.23)
*PNPLA3* [G]- Weight gain+	0.0133	8.15 (1.54–43.02)
*PNPLA3* [G]+ Weight gain+	3.3×10^−5^	12.00 (3.71–38.79)
	Overweight (BMI 23.0–24.9) (n = 170)	
Category	*P*-value	Crude OR (95%CI)
*PNPLA3* [G]- Weight gain-	-	1.00 (Reference)
*PNPLA3* [G]+ Weight gain-	0.0151	6.79 (1.44–31.89)
*PNPLA3* [G]- Weight gain+	0.0217	7.33 (1.33–40.20)
*PNPLA3* [G]+ Weight gain+	0.0008	13.40 (2.92–61.36)
	Obese (BMI>25.0) (n = 198)	
Category	*P*-value	Crude OR (95%CI)
*PNPLA3* [G]- Weight gain-	-	1.00 (Reference)
*PNPLA3* [G]+ Weight gain-	0.4177	0.46 (0.07–2.99)
*PNPLA3* [G]- Weight gain+	0.8771	0.86 (0.14–5.19)
*PNPLA3* [G]+ Weight gain+	0.4045	2.11 (0.36–12.28)

OR: odds ratio, CI: confidence interval, *PNPLA3*: patatin-like phospholipase domain-containing protein 3, Weight gain: weight gain ≥10 kg after age 20. The risk allele is shown in brackets.

## Discussion

The highlight of this study is that the prevalence of NAFLD is more affected by *PNPLA3* rs738409 polymorphism in non-obese individuals than in obese individuals. In other words, whereas obesity is itself a stronger risk factor for NAFLD, the G risk allele carriers may have a significantly increased risk for NAFLD in case they are non-obese. In addition, the hereditary factor *PNPLA3* rs738409 confers a susceptibility to NAFLD in combination with an acquired factor weight gain in adulthood in non-obese individuals.

In East Asia, NAFLD in non-obese populations has been a matter of concern [2, 3]. According to a recent review, the prevalence of NAFLD in non-obese Asians is approximately 11–21% [3], and therefore, the result of this study was in agreement with this review. Recently, GWAS determined that genetic polymorphisms in *PNPLA3* rs738409 are associated with NAFLD even after the results were adjusted for BMI and metabolic values [9, 10]. The majority of Hispanic people carry the risk allele of G in *PNPLA3* rs738409 [9, 21, 22]. As for East Asia, these polymorphisms were also shown to be associated with NAFLD in Japanese, Taiwanese and Chinese individuals [11, 12, 23, 24]. In reality, the G risk allele frequency is 0.34 in Han Chinese, 0.44 in Japanese healthy controls, 0.60 in Japanese NAFLD patients, 0.22 in European, and 0.12 in African [9, 10, 25]. In the Korean study population, the G risk allele frequency is 0.45 in the healthy controls and 0.56 in NAFLD patients [26]. Therefore, higher frequency of the G allele in *PNPLA3* rs738409 may confer much susceptibility to NAFLD in non-obese individuals especially in Eastern Asians compared with Europeans and Africans. The mechanism how *PNPLA3* rs738409 gene variation affects susceptibility to NAFLD in non-obese individuals has not yet been completely elucidated. According to the previous reports, the *PNPLA3* rs738409 variant may affect susceptibility to NAFLD via some plausible mechanisms, including impairment of intrahepatocellular lipolysis [27], lipid droplets remodeling [28], very low-density lipoprotein (VLDL) secretion [29], and retinol metabolism via retinyl-palmitate lipase activity [30] and the G risk allele in *PNPLA3* rs738409 is a risk factor for NAFLD, especially in subjects without metabolic syndrome [31]. Altogether, considering the relatively high frequency of G risk allele in *PNPLA3* rs738409 in Japanese, we suggested that the *PNPLA3* rs738409 polymorphism may play a significant role in NAFLD in non-obese Japanese individuals.

As previously described in a meta-analysis, a *PNPLA3* rs738409 variant was associated with the severity of NAFLD in populations across the world [32] and with the development of advanced hepatic fibrosis in Japanese [24]. Shen et al. reported that NAFLD patients carrying *PNPLA3* rs738409 GG genotype have more sensitivities to the interventional effects of lifestyle modification [33]. Consequently, considering the high mortality of lean patients with NAFLD [8], public information to help people avoid weight gain in adulthood in order to prevent NAFLD in non-obese populations is important.

In this study, the genetic polymorphisms of *NCAN* rs2228603, *GCKR* rs780094, *PPP1R3B* rs4240624 and *LYPLAL1* rs12137855 were not associated with NAFLD in contrast to the results of previous European and American studies [21, 22]. As for *GCKR* rs780094, East Asians studies have shown that this genotype was associated with NAFLD [24, 34, 35, 36]. Tan et al. demonstrated that the association between *GCKR* rs780094 and NAFLD is significant in the Indians but not significant in Malays and Chinese [37]. One recent meta-analysis including 2091 NAFLD patients and 3003 normal controls indicated that *GCKR* rs780094 is significantly associated with NAFLD in both Asians and other ethnicity [38]. Therefore, further examinations are necessary to determine whether this discrepancy is explained by ethnicity or the sample size of the study population.

Interestingly, *CYP17A1* rs1004467 was mildly associated with NAFLD in normal weight individuals. *CYP17A1* gene encodes a key enzyme in the metabolism of steroid which influences hypertension, VFA and SFA in Japanese women [18]. A previous report suggested that visceral fat accumulation is an important factor in the development of NAFLD in Japanese individuals [39]. Taken together, we suggest that *CYP17A1* rs1004467 is involved in the pathogenesis of NAFLD via visceral fat accumulation in a Japanese population of normal weight. To our knowledge, there is no article that describes the association between *CYP17A1* rs1004467 and NAFLD, so further research will be necessary in the future.

Several limitations of this study are noted. First, fatty liver was diagnosed by US. US is currently the most common method for assessing hepatic steatosis [40], whereas it is insensitive to hepatic steatosis when there is <20% fat [41]. Although liver biopsy is the gold standard for the diagnosis of NAFLD, it is not suitable from a safety standpoint at health checkups. Proton magnetic resonance spectroscopy is not practical for a financial reason [40]. Second, a majority of the participants are likely vigilant with regard to their health [42], the possibility of examinee bias cannot be denied. Third, nutritional intake is linked with fatty liver [43, 44], but the assessment of diet was not included in the questionnaire given during routine checkups [13]. Fourth, information on insulin resistance was unavailable in this study, although it has been closely linked to NAFLD [5, 39, 45]. Currently, an insulin resistance marker such as the homeostasis model assessment ratio (HOMA-R) is not investigated in routine checkups. Lastly, this study did not support the previous report which indicated the linkage between the PNPLA3 rs738409 genetic polymorphism and advanced hepatic fibrosis in patients with NAFLD [24]. The values of FIB4 index were low in this study, and so we suggested that there were few patients with severe NASH at our health checkup compared with the hospital based study [24].

In summary, these findings have demonstrated the novel idea that *PNPLA3* rs738409 may robustly affect the pathogenesis of NAFLD in non-obese individuals compared with obese individuals and may have an additive joint effect along with changes in lifestyle such as weight gain ≥10 kg after age 20. Further investigations are required to elucidate the clinical significance of the genetic influences on NAFLD in non-obese populations across East Asia.

## Supporting Information

S1 Table SNP-specific primers and probes for PCR amplification. *NCAN*, neurocan; *LYPLAL1*, lysophospholipase-like 1; *GCKR*, glucokinase regulatory protein; *PPP1R3B*, protein phosphatase 1.
(DOCX)

S2 Table Questionnaire for a specific health checkup in Japan. (DOCX)

S3 Table The demographic, clinical and metabolic features of NAFLD and non-NAFLD. BMI, body mass index; WC, Waist circumference; SBP, systolic blood pressure; DBP, diastolic blood pressure; AST, aspartate aminotransferase; ALT, alanine aminotransferase; GGT, gamma-glutamyl transpeptidase; TC, total cholesterol; TG, triglyceride; HDL, high density lipoprotein- cholesterol; LDL, low density lipoprotein-cholesterol; FPG, fasting plasma glucose; T2D, type 2 diabetes. Continuous values are expressed as the means ±SD. *P-*values were calculated with the Mann-Whitney *U* test for continuous variables and the chi square test for categorical variables.
(DOCX)
