# Supplementary material for: The Impact of PNPLA3 rs738409 Genetic Polymorphism and Weight Gain ≥10 kg after Age 20 on Non-Alcoholic Fatty Liver Disease in Non-Obese Japanese Individuals
Source: PLoS One. 2015 Oct 20;10(10):e0140427. doi: 10.1371/journal.pone.0140427 (PMC4617644; doi:10.1371/journal.pone.0140427)
Supplement: S1 Table — NCAN, neurocan; LYPLAL1, lysophospholipase-like 1; GCKR, glucokinase regulatory protein; PPP1R3B, protein phosphatase 1. (DOCX) [file pone.0140427.s001.docx]

**S1 Table.**

| Gene | SNP | Direction | Sequence |
| --- | --- | --- | --- |
| *NCAN* | rs2228603 | Forward | TGCCCCTCGGATAAAGTGGA |
|  |  | Reverse | TAGGAAGGCAGTGACACTCG |
|  |  | Probe-1 | GACTTGCCCATCCTGGT-Fluorescein |
|  |  | Probe-2 | LC Red 640-GCCAAGGACAATGTCGTGAGGGT-Phosphate |
| *LYPLAL1* | rs12137855 | Forward | AGTATCACCCAATCAATACGAG |
|  |  | Reverse | GGAAGAGAGAGGAGAATAGAATATG |
|  |  | Probe-1 | GTTCCTATTGTCCCTTCAGTCTGATGAAAATCT-Fluorescein |
|  |  | Probe-2 | LC Red 640-GACATAAACCTAAACGAAAATAC-Phosphate |
| *GCKR* | rs780094 | Forward | TCCACTAAACCACAGGCTTC |
|  |  | Reverse | CCCGGCCTCAACAAATG |
|  |  | Probe-1 | GCCCCAGTTTTTTAGACCATGACTG-Fluorescein |
|  |  | Probe-2 | LC Red 640-CACATGTTTGCTGATCA-Phosphate |
| *PPP1R3B* | rs4240624 | Forward | GCGTTACCTTCCCTGTGAAT |
|  |  | Reverse | ACTGTAGGATGACTGTAGTT |
|  |  | Probe-1 | GATGTGACAGATATGCTAATTATCCCGATCTG-Fluorescein |
|  |  | Probe-2 | LC Red 640-TCACCATACCTTCTATGTATCAA-Phosphate |
