# Supplementary material for: The Impact of PNPLA3 rs738409 Genetic Polymorphism and Weight Gain ≥10 kg after Age 20 on Non-Alcoholic Fatty Liver Disease in Non-Obese Japanese Individuals
Source: PLoS One. 2015 Oct 20;10(10):e0140427. doi: 10.1371/journal.pone.0140427 (PMC4617644; doi:10.1371/journal.pone.0140427)
Supplement: S2 Table — (DOCX) [file pone.0140427.s002.docx]

**S2 Table.**

Please complete in your own handwriting. Please select and circle your answer for each of the following questions.

NO Questions

Q1-3. Are you currently taking the following medications?

Answer choices

Q1. a drug to lower blood pressure ① Yes ② No

Q2. insulin injections or a drug to lower blood glucose ① Yes ②

Q3. a drug to lower cholesterol ① Yes ② No

Q4. Have you ever been told by a doctor that you have stroke (e.g., cerebral hemorrhage, cerebral

infarction) or have you ever received treatment for stroke? ① Yes ② No

Q5. Have you ever been told by a doctor that you have heart disease (e.g., angina, myocardial infarction)

or have you ever received treatment for heart disease? ① Yes ② No

Q6. Have you ever been told by a doctor that you have chronic renal failure or have you ever received

treatment for chronic renal failure (dialysis)? ① Yes ② No

Q7. Have you ever been told by a doctor that you have anemia? ① Yes ② No

Q8. Are you a current regular smoker?

(“current regular smoker” is a person who has smoked a total of 100 or more cigarettes or

smoked for 6 months or longer and has been smoking for the last 1 month.) ① Yes ② No

Q9. Have you gained ≥10 kg since you were 20 years old? ① Yes ② No

Q10. Have you been exercising at least 2 days per week, at least 30 minutes each at an intensity that causes

a slight sweat, for at least 1 year? ① Yes ② No

Q11. Do you walk for at least 1 hour everyday or have equivalent physical activities in your daily life? ① Yes ② No

Q12. Do you walk faster than people of your age and gender? ① Yes ② No

Q13. Have you had a weight gain or loss of ≥3 kg over the last year? ① Yes ② No

Q14. How fast do you eat compared to others? ① Faster ② Normal or slower

Q15. Do you have an evening meal within 2 hours before going to bed 3 days or more per week? ① Yes ② No

Q16. Do you eat after the evening meal (have a fourth meal) 3 days or more per week? ① Yes ② No

Q17. Do you skip breakfast 3 days or more per week? ① Yes ② No

Q18. How much alcohol do you drink per day?

(20g of alcohol is equivalent to 120ml of Japanese sake, 400ml of beer, 80ml of shochu (alcohol content 25%), 50ml of whisky, and 160ml of wine) ①<20g ② 20-30g ③ 30-60g ④ >60g ⑤ Rarely (can’t drink)*

Q19. How many times a week do you drink alcohol?

Q20. Do you feel refreshed after a night’s sleep? ① Yes ② No

* Q18 and Q19 were modified from the original edition [13] to diagnose NAFLD accurately [7]. Q8-Q20 were defined as lifestyle items.
