# Supplementary material for: The Impact of PNPLA3 rs738409 Genetic Polymorphism and Weight Gain ≥10 kg after Age 20 on Non-Alcoholic Fatty Liver Disease in Non-Obese Japanese Individuals
Source: PLoS One. 2015 Oct 20;10(10):e0140427. doi: 10.1371/journal.pone.0140427 (PMC4617644; doi:10.1371/journal.pone.0140427)
Supplement: S3 Table — BMI, body mass index; WC, Waist circumference; SBP, systolic blood pressure; DBP, diastolic blood pressure; AST, aspartate aminotransferase; ALT, alanine aminotransferase; GGT, gamma-glutamyl transpeptidase; TC, total cholesterol; TG, triglyceride; HDL, high density lipoprotein- cholesterol; LDL, low density lipoprotein-cholesterol; FPG, fasting plasma glucose; T2D, type 2 diabetes. Continuous values are expressed as the means ±SD. P-values were calculated with the Mann-Whitney U test for continuous variables and the chi square test for categorical variables. (DOCX) [file pone.0140427.s003.docx]

**S3 Table.** The demographic, clinical and metabolic features of NAFLD and non-NAFLD.

| Variable | Underweight (BMI<18.5) (n=65) | | Normal weight (BMI 18.5-22.9) (n=391) | | | | Overweight (BMI 23.0-24.9) (n=170) | | | Obese (BMI >25.0) (n=198) | | |
| --- | --- | --- | --- | --- | --- | --- | --- | --- | --- | --- | --- | --- |
|  | Non-NAFLD(n=65) | | | NAFLD (n=60) | Non-NAFLD (n=331) | *P*-value | NAFLD (n=70) | Non-NAFLD (n=100) | *P*-value | NAFLD  (n=142) | Non-NAFLD  (n=56) | *P*-value |
| Age (years) | 56.7±8.9 | 56.3±11.4 | | | 54.0±10.3 | 0.1322 | 54.5±9.8 | 55.1±10.6 | 0.7901 | 53.6±8.9 | 51.9±9.7 | 0.2333 |
| Gender (male) | 17 (26.1%) | 49 (81.6%) | | | 193 (58.3%) | 6.0×10^-4^ | 64 (91.4%) | 69 (69.0%) | 4.8×10^-4^ | 117 (82.3%) | 39 (69.6%) | 0.0481 |
| BMI (kg/m^2^) | 17.4±0.8 | 21.7±0.9 | | | 20.8±1.2 | 7.3×10^-7^ | 23.9±0.5 | 23.8±0.5 | 0.2873 | 27.9±2.6 | 26.8±1.9 | 0.0034 |
| WC (cm) | 69.8±4.7 | 82.5±4.1 | | | 77.8±4.9 | 1.1×10^-10^ | 87.0±3.8 | 84.3±4.1 | 5.9×10^-5^ | 94.1±6.2 | 90.9±6.6 | 2.7×10^-4^ |
| SBP (mmHg) | 111.4±17.3 | 120.5±19.3 | | | 116.5±16.3 | 0.2266 | 122.6±15.9 | 122.6±15.5 | 0.9079 | 125.8±12.0 | 120.6±17.1 | 0.0049 |
| DBP (mmHg) | 67.4±9.8 | 75.8±12.3 | | | 71.3±10.5 | 0.0202 | 76.9±8.8 | 75.1±10.6 | 0.1279 | 79.0±9.6 | 74.0±10.2 | 0.0017 |
| AST (IU/l) | 21.9±5.7 | 24.1±7.5 | | | 21.1±5.1 | 0.0034 | 24.7±8.1 | 21.6±5.2 | 0.0016 | 28.4±11.6 | 21.5±6.6 | 1.4×10^-6^ |
| ALT (IU/l) | 16.4±5.4 | 26.6±13.8 | | | 17.9±7.0 | 4.8×10^-7^ | 29.2±17.5 | 20.4±9.1 | 2.6×10^-8^ | 37.9±23.2 | 20.6±10.9 | 7.6×10^-11^ |
| GGT (IU/l) | 19.8±11.1 | 35.1±44.7 | | | 27.2±23.6 | 3.2×10^-4^ | 40.6±34.2 | 28.8±21.7 | 1.9×10^-4^ | 44.5±41.1 | 32.8±34.0 | 2.3×10^-6^ |
| FIB4 index |  | 1.29±056 | | |  |  | 1.18±0.53 |  |  | 1.19±0.53 |  |  |
| FIB4 index <1.3 |  | 38 (63.3%) | | |  |  | 51 (72.9%) |  |  | 96 (67.6%) |  |  |
| FIB4 index 1.3-2.67 |  | 22 (36.7%) | | |  |  | 18 (25.7%) |  |  | 41 (28.9%) |  |  |
| FIB4 index >2.67 |  | 0 (0.0%) | | |  |  | 1 (1.4%) |  |  | 5 (3.5%) |  |  |
| TC (mg/dl) | 209.6±29.7 | 211.3±31.8 | | | 201.7±31.9 | 0.0385 | 202.8±38.2 | 206.3±34.8 | 0.4751 | 199.9±31.8 | 203.4±37.9 | 0.5336 |
| TG (mg/dl) | 67.2±29.2 | 132.7±61.0 | | | 84.6±48.8 | 1.4×10^-12^ | 120.8±65.0 | 95.1±48.4 | 0.0076 | 131.7±70.2 | 91.7±33.6 | 1.9×10^-5^ |
| HDL (mg/dl) | 81.4±18.3 | 54.2±11.3 | | | 67.0±15.9 | 1.3×10^-9^ | 54.6±12.4 | 61.6±13.1 | 6.1×10^-4^ | 52.1±12.3 | 60.0±15.1 | 3.9×10^-4^ |
| LDL (mg/dl) | 118.8±24.0 | 137.8±26.7 | | | 122.3±27.7 | 1.4×10^-4^ | 131.1±35.8 | 130.2±31.6 | 0.9886 | 129.2±28.5 | 128.7±34.4 | 0.6815 |
| FPG (mg/dl) | 94.2±16.0 | 103.6±24.0 | | | 96.4±12.7 | 0.0057 | 105.3±21.4 | 98.6±13.2 | 0.0178 | 109.4±28.2 | 101.5±18.6 | 0.0274 |
| Hypertension | 6 (9.2%) | 21 (35.0%) | | | 62 (18.7%) | 0.0046 | 23 (32.8%) | 36 (36.0%) | 0.6718 | 58 (40.8%) | 12 (7.1%) | 0.0101 |
| T2D | 5 (7.6%) | 6 (10.0%) | | | 13 (3.9%) | 0.0543 | 5 (7.1%) | 4 (4.0%) | 0.2870 | 27(19.0%) | 4 (3.9%) | 0.0481 |
| Dyslipidemia | 25 (38.4%) | 42 (70.0%) | | | 139 (41.9%) | 6.2×10^-5^ | 48 (68.5%) | 55 (55.0%) | 0.0747 | 93 (65.4%) | 27 (48.2%) | 0.0250 |
| Weight gain ≥10 kg after age 20 | 1 (1.5%) | 19 (31.6%) | | | 37 (11.1%) | 3.1×10^-5^ | 47 (67.1%) | 44 (44.0%) | 0.0029 | 126 (88.7%) | 41 (73.2%) | 0.0068 |
